# Something is not nothing: Hair-tested substance use and cognitive functions in a large community sample of young adults

**DOI:** 10.1192/j.eurpsy.2026.10156

**Published:** 2026-01-29

**Authors:** Lukas Eggenberger, Clarissa Janousch, Lydia Johnson-Ferguson, Markus R. Baumgartner, Tina M. Binz, Denis Ribeaud, Manuel Eisner, Lilly Shanahan, Boris B. Quednow

**Affiliations:** 1 Experimental Pharmacopsychology and Psychological Addiction Research, Department of Adult Psychiatry and Psychotherapy, University Hospital of Psychiatry Zurich, University of Zurich, Zurich, Switzerland; 2 Jacobs Center for Productive Youth Development, University of Zurich, Zurich, Switzerland; 3 Digital Society Initiative, University of Zurich, Zurich, Switzerland; 4 Department of Global Public Health, Karolinska Institute, Stockholm, Sweden; 5 Forensic Pharmacology and Toxicology, Institute of Forensic Medicine, University of Zurich, Zurich, Switzerland; 6 Institute of Criminology, University of Cambridge, Cambridge, United Kingdom; 7 Department of Psychology, University of Zurich, Zurich, Switzerland; 8 Neuroscience Center Zurich, University of Zurich and Swiss Federal Institute of Technology, Zurich, Switzerland

**Keywords:** addiction, ecstasy, hair toxicology, neuropsychology, stimulants, substance use disorder

## Abstract

**Background:**

Substance use has consistently been linked with cognitive impairments. However, most previous studies have focused on highly selective samples of individuals with chronic substance use disorders and have typically relied solely on self-reports. The associations between recreational use patterns of single or multiple substances and cognitive functioning in representative samples remain unclear.

**Methods:**

We measured over 100 substances and their metabolites over the past 3 months in 850 young adults (48.6% female, *M*_age_ = 24.4) from a community-based cohort, using quantitative hair analysis. We assessed sustained attention, working memory, declarative memory, and a total cognitive performance index using the Cambridge Neuropsychological Test Automated Battery. We regressed cognition on hair substance concentrations, adjusting for sex, household socioeconomic status, migration background, education, gaming experience, and self-reported daily tobacco and alcohol use.

**Results:**

In their hair samples, 386 (45.5%) participants tested positive for at least one psychotropic substance other than alcohol and nicotine. Higher hair concentrations of Δ9-tetrahydrocannabinol (Cohen’s *d* = 0.40) and codeine (*d* = 0.22) were associated with lower sustained attention; higher concentrations of ketamine (*d* = 0.59) with worse declarative memory. Higher hair concentrations of cocaine and a higher polysubstance use severity index (PSUSI) were associated with both reduced attention (cocaine: *d* = 0.21; PSUSI: *d* = 0.30) and declarative memory (cocaine: *d* = 0.20; PSUSI: *d* = 0.29).

**Conclusions:**

In this community sample of young adults, substance use was highly prevalent and associated with reduced cognitive performance, with small-to-moderate effect sizes. Cognitive consequences of recreational substance use may have been previously underestimated.

## Introduction

The prevalence of illicit and nonmedical substance use typically peaks during adolescence or young adulthood [1, 2]. These developmental periods are considered highly sensitive periods for brain maturation in the prefrontal cortical regions. These regions play a central role in high-level cognitive processes and continue to develop well into the mid-twenties [3–5]. Disruptions in their development can result in impaired cognitive functions such as attention and memory, skills that are critical for effective daily and occupational functioning. Indeed, the use of illicit substances during these developmental periods has been linked to various functional impairments. For example, frequent cannabis use during adolescence negatively affects intellectual and educational performance [6, 7]. However, the cognitive consequences of exposure to the full spectrum of recreational substances in young adulthood are not fully understood. This study investigates the associations between cognitive functions and the most commonly used substances, such as cannabis, cocaine, 3,4-methylenedioxymethamphetamine (MDMA, “Ecstasy”), opioids, ketamine, and dextromethorphan (DXM), which were measured by toxicological hair testing.

### Cognitive effects of cannabis use

Cannabis is the most used illicit substance worldwide [8]. Its main psychoactive compound, Δ9-tetrahydrocannabinol (THC), remains in the human body for a relatively long time [9]. Accordingly, it can cause both acute and post-acute effects on cognitive functions. Small-to-moderate doses of experimentally administered THC have been found to acutely reduce verbal learning and memory performance [10]. Meta-analyses of cross-sectional studies have consistently identified small-to-moderate associations between the post-acute effects of chronic cannabis use and reduced attention, memory, and executive function [11, 12]. In turn, these functions improve with length of abstinence [13, 14].

### Cognitive effects of cocaine use

The global supply and demand for cocaine are currently at record levels [15]. Since 2016, wastewater analyses in European cities have documented a steady increase in the concentration of benzoylecgonine, the main excreted metabolite of cocaine [16]. Similar trends have been observed in Australia and Brazil [15]. These trends are particularly concerning because both cocaine addiction [17, 18] and regular recreational cocaine use have been associated with reduced cognitive functions, specifically attention, working memory, and declarative memory [19]. Neurodevelopmental impairments following cocaine exposure during adolescence have been reported in rodent models [20–22]. Moreover, a younger age of onset of cocaine use has been associated with greater cognitive impairments in a large clinical sample of cocaine users confirmed by hair testing [19].

### Cognitive effects of substituted amphetamines

Ecstasy, which typically contains the substituted amphetamine derivative MDMA, is another commonly used substance among young adults [8, 23]. Previous research has reported cross-sectional associations between frequent self-reported and hair-tested MDMA use and chronic impairments, particularly in declarative memory [24–26]. Similarly, a few small longitudinal studies have suggested that MDMA use induces memory deficits in novice users that persist after long-term abstinence, possibly because of its neurotoxic effects [27–29]. Regarding the non-medical use of methamphetamine, inhibitory control has been identified as the most strongly affected cognitive domain in young users [30]. Notably, among individuals with methamphetamine use disorder, deficits in attention, executive functioning, as well as visual learning and memory have been observed [31]. Finally, in rodents, adolescent exposure to amphetamine-type stimulants, such as MDMA, amphetamine, and methamphetamine, is associated with worse learning and working memory [32].

### Cognitive effects of prescription opioids, dissociatives, and polysubstance use

Nonmedical use of prescription opioids, such as opioid painkillers (e.g., morphine, oxycodone) and cough syrup (e.g., codeine), has recently reached epidemic proportions in the US [33] and has increased steadily in Europe [8]. However, the effects of chronic nonmedical use of prescription opioids on cognition are poorly understood. To date, a single study employing hair testing has suggested that frequent nonmedical opioid use is associated with reduced attention and declarative memory performance [34].

Studies of cognitive impairments associated with less frequently used substances are also limited. For example, chronic ketamine use has been linked with impairments in several memory functions and attention [35, 36], which appear to be more pronounced with an adolescent onset of use [37]. Systematic studies examining the chronic effects of DXM use on cognition are lacking, but one case report suggested potential neuropsychological deficits after long-term use [38]. Finally, there is limited research on the cognitive consequences of polysubstance use, despite its high prevalence in early adulthood [39]. However, preliminary data suggest that polysubstance use, including stimulants or MDMA, is associated with widespread cognitive deficits affecting attention, working memory, declarative memory, and socio-cognitive functioning [25, 40].

### Limitations of previous studies

Previous research on substance use and cognitive functions has largely focused on small samples of individuals with chronic and regular use patterns. While these studies offer valuable insights into high-risk populations, they are susceptible to selection bias [41] and represent only a small fraction of the overall user population. Casual or recreational use, which is far more prevalent, has been investigated less frequently, particularly in community-based samples. Moreover, most prior studies relied on self-reported substance use measures, often of questionable validity [42], or have focused on a narrow range of single substances, even though polysubstance use is now the norm rather than the exception [43].

In addition, most studies have adjusted for only a limited set of confounders, such as age, sex, and education, without accounting for broader sociodemographic or lifestyle factors that significantly influence cognitive functioning at the population level. Research on age-related cognitive declines, for example, underscores the importance of socioeconomic status (SES) and migration background (e.g., [42–44]). These covariates should also be considered in studies of adolescents and young adults [45–47]. Furthermore, in the context of computerized tests of cognitive functions, prior experience with specific tasks, such as playing action video games, may enhance performance (see, e.g., the meta-analysis by [48]), which is particularly relevant for younger age groups. Finally, lifestyle factors such as smoking [49–52] and frequent alcohol use [53–55] have been consistently linked with cognitive performance.

### Aim of the present study

For years, there have been calls for more evidence-based drug policies [56]. The cognitive consequences of substance use among adolescents and young adults are an important part of this evidence, especially in view of the global increase in use among these age groups [57] and the ongoing debate on the legalization of cannabis and other substances [58]. The aim of this study was to investigate associations of substance use and cognitive performance in a large community study of young adults with known high prevalence and intensity of use of a broad range of substances [59]. To objectively confirm and quantify substance use, we used hair testing, which is sensitive to many compounds and enabled us to detect occasional or recreational use of substances (except for cannabis, for which hair testing typically captures highly regular use only [42, 60]). In addition to single substances, we measured polysubstance use. Finally, we adjusted for several known confounders in multivariable regression models, such as sex, SES, migration background, education, gaming experience, and tobacco and alcohol use.

Based on previous findings, we expected that (1) regular cannabis use is associated with decreased attention and declarative memory [11–13]; (2) cocaine use is associated with decreased attention, working memory, and declarative memory [17–19]; (3) MDMA use is associated with decreased declarative memory [24–26]; (4) prescription opioid use is associated with decreased attention and declarative memory [34]; (5) ketamine use is associated with decreased declarative memory [35, 36]; (6) DXM use is associated with decreased working and declarative memory [38]; and (7) polysubstance use is associated with decreased attention, working memory, and declarative memory (38).

## Methods and materials

### Participants

We used data from the *Zurich Project on Social Development from Childhood to Adulthood* (z-proso), a prospective, longitudinal, community-based cohort study initiated in 2004 to investigate the social, emotional, and behavioral development of children and to identify risk and protective factors for antisocial behavior and mental health across the life course [61]. The cohort was designed to be representative of children entering first grade in the city of Zurich in August 2004 (target sample *N* = 1,675). For the current analysis, no exclusion criteria were applied beyond the availability of valid hair samples and cognitive test data in the ninth assessment wave (W9) when participants were 24 years old (*M* = 24.5, *SD* = 0.4, range = 23.1 to 25.9). The absence of additional exclusion criteria ensured that the community population was represented as closely as possible.

**Figure 1.**
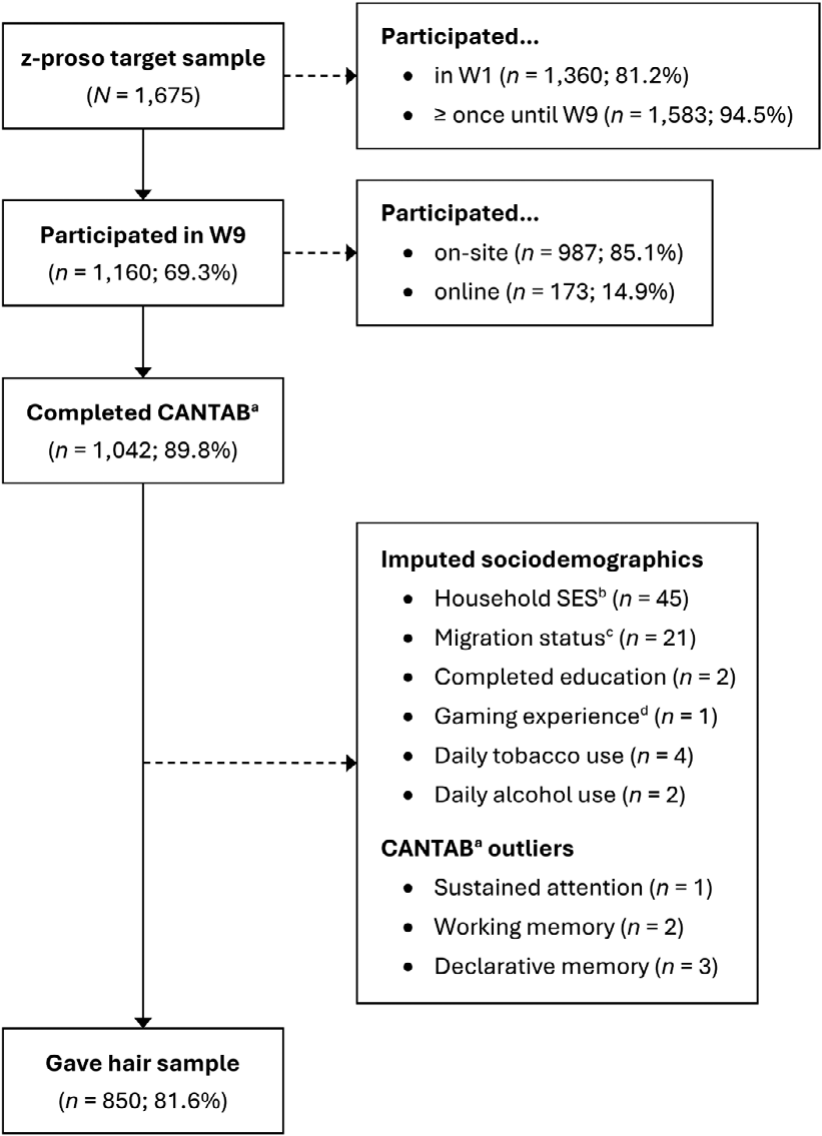
Flow diagram for study sample (*n* = 850). *Note.* N/n = number of participants. W1 – W9 = assessment waves, ranging from age 7 (W1) to age 24 (W9). ^a^ Cambridge Neuropsychological Test Automated Battery. ^b^ Socioeconomic status assessed with the International Socio-Economic Index of Occupational Status, ranging from 14 (unskilled worker) to 90 (judge). ^c^ Positive migration background if both parents were not born in Switzerland. ^d^ Frequency of playing action-packed video games in the past year, averaged across ages 20 and 24.

Of the 1,160 participants assessed in W9, 1,042 (89.8%) completed the cognitive test battery, 887 (76.5%) provided a hair sample, and 850 (73.3%) both, completed the cognitive tests and provided a hair sample (Figure 1). Six participants in the cognitive test sample (*n* = 1,042) had outliers, which were replaced by missing values (Supplementary Figure S1). A random forest algorithm was used to impute the sociodemographic information of 71 participants. Information on sample agreement rates is provided in Supplementary Text S1.

The authors assert that all procedures contributing to this work comply with the ethical standards of the relevant national and institutional committees on human experimentation and with the Helsinki Declaration of 1975, as revised in 2013. All procedures involving human subjects were approved by the Cantonal Ethics Committee Zurich (BASEC #2017-02021) and the Ethics Committee of the Faculty of Arts and Social Sciences of the University of Zurich. Written informed consent was obtained from all participants. Participants received monetary compensation for completing the survey, the cognitive tests, and for providing a hair sample (on-site: 150 CHF, 50 CHF, and 30 CHF; online: 100 CHF, 40 CHF, and 50 CHF, respectively).

### Measures

#### Sociodemographic and lifestyle factors

Information was collected on participants’ sex assigned at birth (0 = *male*, 1 = *female*); the SES of the household in which they grew up (ranging from 16 = *unskilled worker* to 90 = *judge*), using the *International Socio-Economic Index of Occupational Status* ([62]); migration background (0 = *at least one parent born in Switzerland*, 1 = *both parents not born in Switzerland*), and highest completed education (0 = *below apprenticeship*, 1 = *apprenticeship*, 2 = *vocational tertiary*, 3 = *academic tertiary*). Participants reported their experience of playing action-packed violent computer or video games in the past year (e.g., first-person shooters; ranging from 1 = *never* to 7 = *daily*) and their daily tobacco and alcohol consumption (dichotomized as 0 = *never or less than daily* and 1 = *daily*). A more detailed overview of these assessments is shown in Supplementary Table S1.

#### Mental health symptoms

To evaluate mental health symptoms, the anxiety-depressive (internalizing) and ADHD (externalizing) symptom scales from the Social Behavior Questionnaire (SBQ; [63]) were used. Participants rated in eight items how often they experienced internalizing symptoms (e.g., “couldn’t enjoy anything”) in the previous month and in four items externalizing symptoms (e.g., “were easily distracted”) in the past 12 months. Responses were provided on a 5-point scale ranging from 1 (never) to 5 (very often). Previous studies have established the SBQ’s adequate psychometric properties in the z-proso study [64].

#### Hair toxicological analysis

The concentration of 114 substances and metabolites in the hair of the participants was determined by a validated liquid chromatography–tandem mass spectrometry (LC–MS/MS) method. After washing (water, acetone, hexane) and extraction, analytes were separated on Kinetex® C18 (THC, CBD, CBN)/F5 columns (Phenomenex, Torrance, CA, USA) and detected in scheduled multiple reaction monitoring mode, using a QTrap 5500 system (Sciex, Marlborough, MA, USA) with atmospheric pressure chemical ionization/electrospray ionization, depending on analyte class. The method was revalidated for selectivity, linearity (r ≥ 0.98), limits of quantification (0.5–100 pg/mg depending on analyte), accuracy and precision (acceptance criteria ±30% RSD for the bias, RSD_R_ ≤ 30%, and RSD_T_ ≤ 30% for the repeatability and the intermediate precision), and robustness (RSD ±30%), according to forensic toxicology guidelines. Full validation data are available elsewhere [65, 66].

Since at least 3 cm of hair was analyzed for each sample, except for eight samples with ≥1.5 cm, the concentrations approximately reflected the cumulative substance exposure over the 3 months prior to the assessment. Importantly, THC in hair can only be detected in the context of regular use [42, 60]. For the present study, we grouped related substances and their metabolites into categories and converted medical opioids into morphine-equivalent concentrations, according to (32) and (65). To avoid power constraints, we only considered substance categories with *n* > 30 positive cases. We also employed a polysubstance use severity index (PSUSI; [40]), which was calculated for each participant by summing tertial scores of hair concentration levels (not detected = 0, low = 1, medium = 2, high = 3) across all substances. For example, a person with a medium level of MDMA [level 2], a high level of cocaine [level 3], and a low level of ketamine [level 1] received a PSUSI of 6. All substance concentrations were log-transformed using the formula log(x + 1) to facilitate linear estimation. An overview of the substance use variables and morphine equivalents (ME) is shown in Supplementary Table S2.

#### Cognitive test battery

We used the *Cambridge Neuropsychological Test Automated Battery* (CANTAB; [67]), which is a fully computerized nonverbal cognitive test battery. We used three tasks: rapid visual processing (measure: A prime) to assess sustained attention, spatial working memory (measure: total errors) to assess visuospatial working memory performance, and paired associates learning (PAL; measure: adjusted total errors) to assess declarative visuospatial memory performance. We inverted the working memory and declarative memory scores so that positive values indicated positive performance. Furthermore, we calculated a total cognitive score by creating a sum score across the three z-standardized and, if applicable, inverted individual tasks.

### Statistical analysis

We regressed hair substance concentrations as a continuous dose variable and all covariates onto the four CANTAB scores, estimating both bivariate and multivariable associations. We also estimated the effect sizes (Cohen’s *d*) for each substance by comparing the marginal mean CANTAB scores of participants without any substance concentration to those with (a) any substance concentration and (b) low, medium, and high concentrations (i.e., concentration levels), based on a tertile split. More details are provided in the Supplementary (Text S2). Finally, we conducted robustness checks by including internalizing and externalizing symptoms as additional covariates to adjust for potential confounding of psychiatric symptoms. Notably, these scales were not part of our a priori covariate set but were included post hoc due to their conceptual relevance to both substance use and cognitive outcomes. All calculations were performed using *R* version 4.3.2 statistical software [68].

## Results

### Sample descriptives

Approximately half of the sample was female (48.6%), and 44.5% had a migration background. Complete descriptives are presented in Table 1.

**Table 1.** Sample characteristics stratified by any kind of illicit substance detected in hair

	Total sample (*n* = 850)	No substance (*n* = 464)	Any substance (*n* = 386)	Effect^f^ [95% CI]	Effect size interpretation
**Female sex**, *n (%)*	413 (48.6)	244 (52.6)	169 (43.8)	.09 [.02, .15]	very small
**Household SES^a^**, *mean (SD)*	47.0 (19.0)	48.0 (18.9)	45.7 (19.0)	−0.12 [−0.26, −0.01]	very small
**Migration background^b^**, *n (%)*	378 (44.5)	199 (42.9)	179 (46.4)	.03 [.00, .10]	–
**Completed education**, *n (%)*					
Below apprenticeship	79 (9.3)	34 (7.3)	45 (11.7)	.07 [.00, .14]	–
Apprenticeship	323 (38.0)	159 (34.3)	164 (42.5)	.08 [.02, .15]	very small
Vocational	219 (25.8)	127 (27.4)	92 (23.8)	.04 [.00, .11]	–
Academic	229 (26.9)	144 (31.0)	85 (22.0)	.10 [.03, .17]	small
**Gaming experience^c^**, *mean (SD)*	2.4 (1.7)	2.1 (1.6)	2.6 (1.8)	0.30 [0.16, 0.43]	small
**Daily tobacco use**, *n (%)*	287 (33.8)	108 (23.3)	179 (46.4)	.24 [.18, .31]	medium
**Daily alcohol use**, *n (%)*	43 (5.1)	20 (4.3)	23 (6.0)	.04 [.00, .11]	–
**CANTAB^d^**, *mean (SD)* [z-score]					
Sustained attention (sensitivity)	0.90 (0.06)	0.91 (0.06)	0.90 (0.05)	−0.24 [−0.38, −0.11]	small
Working memory (errors)	7.24 (7.81)	6.67 (7.30)	7.92 (8.34)	0.16 [0.02, 0.30]	very small
Declarative memory (errors)	6.41 (7.83)	5.91 (7.19)	7.00 (8.51)	0.14 [0.01, 0.27]	very small
**SBQ^e^**, *mean (SD)*					
Internalizing symptoms	2.51 (0.82)	2.44 (0.78)	2.59 (0.87)	−0.18 [−0.32, −0.05]	very small
Externalizing symptoms	2.83 (0.78)	2.74 (0.76)	2.94 (0.79)	−0.26 [−0.39, −0.12]	small

*Note. n* = number of participants.

a Socioeconomic status assessed with the International Socio-Economic Index of Occupational Status, ranging from 14 (unskilled worker) to 90 (judge).

b Positive migration background if both parents were not born in Switzerland.

c Frequency of playing action-packed video games in the past year, averaged across ages 20 and 24.

d Cambridge Neuropsychological Test Automated Battery.

e Social Behavior Questionnaire.

f Cohen’s *d* for numeric and Cramer’s V for nominal variables, with two-sided 95% confidence interval (CI).

**Figure 2.**
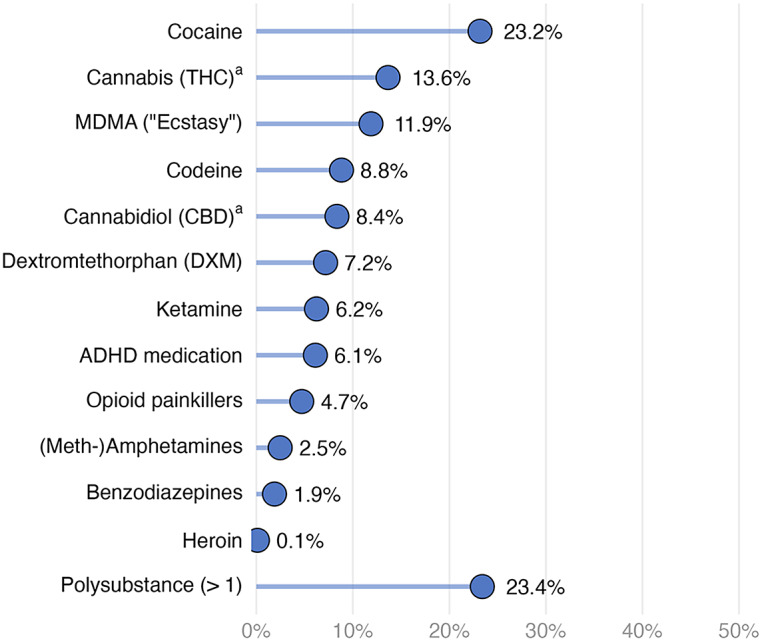
Prevalence of substances in hair (*n* = 850). *Note.* Polysubstance means at least two different substances being detected in hair. Substances with *n* < 30 (i.e., (meth-)amphetamines, benzodiazepines, and heroin) were not included in the analyses due to power constraints. ^a^ Prevalence rates for THC and CBD reflect frequent (weekly+) use.

Figure 2 shows that cocaine was the most prevalent (23.2%) illegal substance detected in hair, followed by THC (13.6%, indicating regular cannabis use only), MDMA (11.9%), and codeine (8.8%). Substances with fewer than 30 positive cases (i.e., amphetamines other than MDMA, benzodiazepines, and heroin) were not included in further analyses due to power constraints. Among substances with at least 30 cases, opioid painkillers had the lowest prevalence rate (4.7%). Approximately a quarter of the participants tested positive for more than one substance (23.4%). The distribution of hair concentrations revealed that most substances were typically used occasionally or recreationally (Supplementary Figure S2), except for THC, where detection in hair reflects regular use.

Bivariate correlations and detailed descriptive statistics of covariates and cognition scores are provided in Supplementary Tables S3 and S4.

### Substance use and cognitive functions

**Figure 3.**
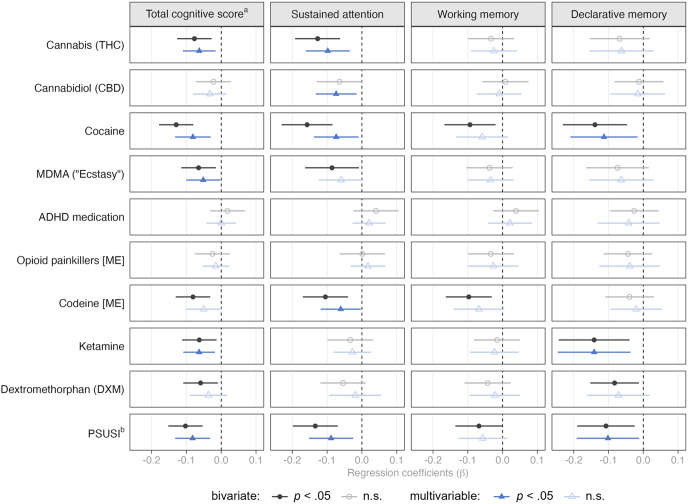
Bivariate and multivariable associations between substance concentrations in hair and cognitive functions. *Note.* Multivariable models include sex, household SES, migration background, education, gaming experience, daily tobacco use, and daily alcohol use as control variables. [ME] = morphine equivalents. ^a^ Total cognitive score reflects mean score of the three CANTAB scores on sustained attention (*z*-standardized), declarative memory (inverted and *z*-standardized), and working memory (inverted and *z*-standardized). ^b^ Polysubstance-Use Severity Index.

Multivariable regression analyses indicated that higher THC concentrations were associated with worse total cognitive performance and worse performance on sustained attention (Figure 3). When examining concentration levels (low/medium/high; Supplementary Table S5), we found that THC concentrations at the lower end – which still indicate regular use – seemed to drive this association. For cocaine, higher concentrations were associated with worse cognitive performance on the total cognitive score, sustained attention, and declarative memory. Particularly, the highest cocaine concentration levels were associated with poorer performance. MDMA concentrations were negatively associated with the total cognitive score only after adjusting for covariates. Here, primarily medium concentration levels were associated with worse performance. Interestingly, the association between medium concentrations of ADHD medications and working memory performance was positive. However, this was only evident when examining concentration levels and not the continuous dose variable.

The association of codeine and sustained attention was negative, mainly driven by high concentration levels. Notably, low doses were associated with worse working memory performance, but only when considering concentration levels and not the continuous dose variable. Ketamine concentrations were negatively associated with total cognitive and declarative memory performance, particularly at low and high concentration levels. The PSUSI was negatively associated with total cognitive performance, sustained attention, and declarative memory, particularly for medium and high PSUSI levels.

In contrast to the multivariable regressions, much stronger effects were observed in the bivariate unadjusted regression models, highlighting the need to adjust for confounders when assessing associations of substance use and cognition. Detailed bivariate and multivariable model estimates are presented in the Supplementary (Figures S3, S4, S5, and S6).

Finally, a higher percentage of participants using substances deviated more than 1.5 or 2 standard deviations from the mean cognitive scores than participants not using substances (Supplementary Table S6). Additional robustness checks, which adjusted the regression models for internalizing and externalizing symptoms, did not significantly change any of the associations between substance concentrations and cognitive functions (Supplementary Figure S7).

## Discussion

### Summary of results

This study examined cross-sectional associations between substance concentrations in hair and cognitive functions in a large community sample of young adults, primarily exhibiting recreational use patterns [59, 69]. Specific substances were associated with distinct cognitive functions: Sustained attention was reduced in users of cannabis, cocaine, and codeine, as well as in young adults with polysubstance use. Declarative visuo-spatial memory was reduced in young adults with cocaine or ketamine use as well as in those with polysubstance use. Total cognition was poorer in young adults with cannabis, cocaine, MDMA, ketamine, and polysubstance use. In contrast, visuo-spatial working memory was largely not associated with substance use after adjusting for covariates.

### Substance-specific associations with cognitive functions

Confirming our first hypothesis, higher THC hair concentrations in presumed regular cannabis users (*n* = 116) were associated with poorer total cognitive performance (any use: *d* = 0.31) and worse attention (*d* = 0.40), which is consistent with recent meta-analyses [13, 14]. The expected association with declarative memory (*d* = 0.20) was not significant, although a meta-analysis found the largest effect sizes in the learning domain and significant results for delayed memory [13]. However, most of the studies included in this meta-analysis tested verbal declarative memory, and very few tested visuo-spatial associative learning, as we did. Given that a study of long-term daily cannabis users showed that verbal memory was significantly more impaired than visuospatial memory (including the PAL) [70], the PAL was likely not the optimal test to reliably detect memory deficits in weekly to daily cannabis users.

Notably, our study identified individuals with cannabis use through hair testing, which primarily detects weekly to daily use [42, 60, 69]. Our marginal means analyses suggest that even low THC concentrations in hair are associated with reduced attention. THC concentrations in hair may be a less reliable reflection of actual cannabis use intensity, which contrasts with other substances measured in hair such as cocaine [71, 72]. Considering the meta-analysis by Scott et al. [13], our findings likely reflect post-acute effects of frequent cannabis use on sustained attention. Reduced attention due to cannabis use is highly relevant in the context of the recent debate on cannabis legalization, especially considering that cannabis legalization in the US has increased the prevalence rates of cannabis use among young adults [73]. These findings underscore the need for a comprehensive prevention and harm reduction strategy within the cannabis legalization discourse, aimed at informing the public and mitigating the potential cognitive consequences of increasing cannabis use among young adults.

Regarding our second hypothesis, higher cocaine hair concentrations among occasional cocaine users (*n* = 197 tested positive) were associated with slightly worse total cognitive performance (any use: *d* = 0.24), attention (*d* = 0.21), and declarative memory (*d* = 0.20), but not with working memory performance (*d* = 0.10). Although previous meta-analyses [14, 18] reported larger effect sizes, the included studies primarily focused on chronic and dependent users with intensive consumption. Similarly, a large study that also investigated recreational users [19] found stronger effect sizes for these cognitive domains (*d* = 0.43–0.44). However, those participants were regular users, averaging 1.1 g of cocaine per week, and exhibited much higher cocaine hair concentrations (mean: 3347 pg/mg), compared to the present sample (mean: 1645 pg/mg). The weaker association with working memory (i.e., only bivariate) may be explained by the fact that working memory deficits are partially reversible with prolonged cocaine abstinence or reduced use [74]. Consequently, occasional users with infrequent consumption may recover their performance. Our results suggest that high cocaine concentrations appear to underlie cognitive performance impairments (Supplementary Table S5), as effect sizes were largest for high users (total cognitive performance: *d* = 0.52, attention: *d* = 0.40, declarative memory: *d* = 0.45, working memory performance: *d* = 0.25) and within the range of previously published effect sizes [14, 18, 19]. While low-to-moderate doses may be less likely to cause clinically significant cognitive impairments, the strikingly high prevalence rate of approximately 23% remains concerning, not only due to cocaine’s high addictive potential but also because of its well-documented adverse effects on mental and physical health [56, 75].

We also hypothesized an association between MDMA use (*n* = 101) and impaired declarative memory performance; however, we observed only an association with total cognition (any use: *d* = 0.25) and a bivariate association with reduced attention (*d* = 0.25), which aligns with findings from a previous meta-analysis [24]. The relatively high prevalence of concurrent use of MDMA with other substances, such as cannabis and cocaine (*n* = 14 pure MDMA users, or 13.9% of all MDMA users), suggests that the attention and global cognitive effects associated with cannabis and cocaine use (see above) may have influenced this population. Moreover, MDMA users in this sample were predominantly rare occasional users, as reflected by the relatively low MDMA hair concentrations (mean 460 pg/mg, median 54 pg/mg). In contrast, previous studies from our laboratory that reported large effects sizes for declarative memory deficits examined individuals with substantially more intensive MDMA use ([25]: mean 3414 pg/mg; [76]: mean 4828 pg/mg; [77] median: 819 pg/mg). Importantly, in MDMA users, verbal declarative memory performance was more strongly affected than visuospatial memory [24, 25]. A verbal memory test would therefore have been more sensitive to detecting MDMA-related memory deficits.

We found a significant association between impaired attention (any use: *d* = 0.22) and intake of codeine (*n* = 75), an opioid commonly found in cough medications, which we analyzed separately from opioid painkillers, such as oxycodone. However, the latter did not show the hypothesized associations with attention or declarative memory. This lack of association may be due to the small number of opioid painkiller users in our sample (*n* = 40), who exhibited only sporadic use (mean ME 154 pg/mg, median ME 20 pg/mg in hair). By contrast, a previous study of prescription opioid users [34] (mean ME 543 pg/mg) reported stronger deficits in attention (Hedge’s *g* = 0.85) and declarative memory (*g* = 0.66). Notably, 52% of participants in that study used codeine or dihydrocodeine, which aligns well with our findings for codeine. Notably, reduced attention was primarily associated with higher codeine concentrations, which are typically required to achieve the euphoric effects linked to codeine use. This suggests that the observed effects may stem from nonmedical use of codeine-containing medications.

We further observed the hypothesized association between ketamine exposure (*n* = 53) and impaired declarative memory (any use: *d* = 0.59), which represented the strongest effect in our sample, despite relatively low hair concentrations. These memory deficits are consistent with previous studies on chronic ketamine users, which reported significant impairments, particularly in tasks involving visual stimulus material [78–80]. In contrast, we found no unique association between DXM exposure (*n* = 61) and cognitive performance after adjusting for covariates. While DXM hair concentrations likely reflect its medical use as a cough suppressant, the primary medical use of ketamine, as an anesthetic or for treatment-resistant depression, are unlikely in this young community sample.

We also observed the expected associations between polysubstance use severity (as measured by the PSUSI) and impairments in total cognition (*d* = 0.21), attention (*d* = 0.17), and declarative memory (*d* = 0.12), but not working memory (*d* = 0.15) after adjusting for covariates. Overall, polysubstance use showed the strongest effects, likely due to its combined number and intensity of different substances. This cumulative exposure may be particularly detrimental to cognitive functioning, even within the recreational and non-dependent use patterns observed in this community sample.

### Sample characteristics

The sample composition and characteristics warrant discussion. First, the prevalence of substance use in this sample is remarkably high, even for Zurich, which typically reports high rates for substances such as cannabis, cocaine, MDMA, and ketamine based on wastewater analyses [16]. The estimated prevalence of cocaine use of approximately 23% is particularly striking. According to recent work [72], a median hair cocaine concentration of 157 pg/mg corresponds to a consumption of roughly 0.13 g/week, whereas the average use in the 95th percentile equals approximately 15.7 g per week. Furthermore, the sample was predominantly well educated, with more than half of participants having completed tertiary education (vocational or academic). Although some self-selection bias may have occurred because participants could choose whether to provide a hair sample, only 10.1% attending the on-site assessment declined to do so.

Notably, although our interpretation assumes that psychoactive substance exposure may contribute to cognitive impairment, the reverse direction cannot be ruled out due to the cross-sectional design of this study. Individuals with pre-existing lower cognitive functioning or executive control may be more prone to substance use or experience greater difficulty regulating consumption [81–84]. Thus, the observed associations may reflect bidirectional or predisposing effects rather than direct consequences of use. Longitudinal and preclinical studies are needed to clarify the temporal sequence and causal pathways underlying these relationships.

### Strengths and limitations

Limitations include, first, the cross-sectional design of the analyses, which does not allow for causal or temporal inferences. Second, we observed mostly small effect sizes for the associations between substance use and cognitive functions, which was expected given the recreational use patterns in this community-based cohort. However, it is important to note that participants’ educational level explained a substantial proportion of the variance in cognitive performance (see, e.g., [46]). Third, the cognitive test battery used in the present study was not exhaustive due to strict time constraints aimed at limiting participant burden, meaning that functions such as processing speed, executive functions, and decision-making – domains shown to be affected in substance-using populations [85] – could not be assessed.

Finally, a major strength of our study is the objective assessment of substance use through hair testing, which is particularly important given the high rates of underreporting of illegal substances, such as cocaine [42]. Additionally, selection bias was minimal, as the original sample was community-representative [61].

## Conclusion

Our results indicate high prevalence rates of substance use among young adults and lower cognitive performance associated with predominantly occasional and recreational use of substances such as cannabis, cocaine, codeine, MDMA, and ketamine, as well as polysubstance use. On the one hand, the high prevalence of substance use in this population likely increases the risk of functional impairments [86, 87], reduced well-being [56, 75], and the development of substance use disorders later in life [88, 89]. On the other hand, the immediate consequences of cognitive dysfunction due to recreational substance use may include impaired driving ability [90], diminished educational or occupational performance, or an increased risk of serious falls, even among younger individuals using opioids [91]. Our findings should be considered in the context of recent discussions about the legalization of substances such as cannabis, providing important evidence to inform drug policy (e.g., [58]). However, further research using longitudinal designs and advanced methods is needed to establish the directionality of these effects and account for additional potential covariates.

## Supporting information

10.1192/j.eurpsy.2026.10156.sm001 Eggenberger et al. supplementary material Eggenberger et al. supplementary material

## Data Availability

Anonymized data, protocols, or other material from earlier project data collections are generally available to the scientific community upon request from author DR (denis.ribeaud@jacobscenter.uzh.ch).

## Supplementary material

The supplementary material for this article can be found at http://doi.org/10.1192/j.eurpsy.2026.10156.
