# Supplementary material for: Something is not nothing: Hair-tested substance use and cognitive functions in a large community sample of young adults
Source: Eur Psychiatry. 2026 Jan 29;69(1):e19. doi: 10.1192/j.eurpsy.2026.10156 (PMC12925671; doi:10.1192/j.eurpsy.2026.10156)
Supplement: Eggenberger et al. supplementary material [file S0924933826101564sup001.docx]

**Supplement**

**Something is not nothing:
Hair-tested substance use and cognitive functions in a large community sample of young adults**

Lukas Eggenberger^1,2,3,*^, Clarissa Janousch^1,2,4^, Lydia Johnson-Ferguson^1,2^,
Markus R. Baumgartner^5^, Tina M. Binz^5^, Denis Ribeaud^2^, Manuel Eisner^2,6^, Lilly Shanahan^2,7,^ *^†^*, Boris B. Quednow^1,2,8,^ *^†^*

*^1^Experimental Pharmacopsychology and Psychological Addiction Research, Department of Adult Psychiatry and Psychotherapy, University Hospital of Psychiatry Zurich, University of Zurich, Zurich, Switzerland*

*^2^Jacobs Center for Productive Youth Development, University of Zurich, Zurich, Switzerland*

*^3^Digital Society Initiative, Zurich, Switzerland*

*^4^Department of Global Public Health, Karolinska Institute, Stockholm, Sweden*

*^5^Center for Forensic Hair Analytics, Zurich Institute of Legal Medicine, University of Zurich, Zurich, Switzerland*

*^6^Institute of Criminology, University of Cambridge, Cambridge, United Kingdom*

*^7^Department of Psychology, University of Zurich, Zurich, Switzerland*

*^8^Neuroscience Center Zurich, University of Zurich and Swiss Federal Institute of Technology, Zurich, Switzerland*

*^†^Contributed equally*

**^*^Corresponding author:** Lukas Eggenberger (lukas.eggenberger@bli.uzh.ch)

**Content**

**S-Texts**

- Text S1: Sample Agreement Rate
- Text S2: Statistical Analysis

**S-Figures**

- Figure S1: CANTAB Outlier Removal
- Figure S2: Distribution of Untransformed vs. Transformed data
- Figure S3: Regression Models: Total Cognitive Score
- Figure S4: Regression Models: Sustained Attention
- Figure S5: Regression Models: Working Memory
- Figure S6: Regression Models: Declarative Memory
- Figure S7: Sensitivity Analyses with Internalizing and Externalizing Symptoms

**S-Tables**

- Table S1: Questions and Items Overview
- Table S2: Substance Groupings and Morphine Equivalents
- Table S3: Descriptives of Study Variables
- Table S4: Correlation Matrix
- Table S5: Marginal Effects of Low, Medium, and High Substance Concentrations
- Table S6: Percentage of Users Scoring 1.5 SD or 2 SD Below Average

**Supplementary References**

**Text S1**

**Sample Agreement Rate**

Women were less likely to provide a hair sample compared to men (22.5% vs. 14.1% not providing a hair sample; *p* < .001). Furthermore, participants with a migration background were less likely to provide a hair sample (22.4% vs. 15.0% not providing a hair sample; *p* = .003). Participants who did not provide a hair sample also had less experience with playing action-packed video games (no hair sample: *M* = 1.9 [*SD* = 1.5]; hair sample: *M* = 2.4 [*SD* = 1.7]; *p* < .001).

We did not find differences in highest completed education between participants who provided a hair sample and those who did not (15.0% below apprenticeship, 16.1% with an apprenticeship, 21.2% with a vocational tertiary education, and 19.9% with an academic tertiary education did not provide a hair sample, respectively; *p* = .276). Similarly, we did not find any differences regarding daily tobacco use (16.1% vs. 19.6% not providing a hair sample; *p* = .201) and daily alcohol use (18.9% vs. 18.4% not providing a hair sample; *p* = .999). Lastly, we did not observe a difference in the total cognitive score between participants who did not provide a hair sample and those did provide a hair sample (no hair sample: *M* = -0.05 [*SD* = 0.71]; hair sample: *M* = 0.01 [*SD* = 0.74]; *p* = .338).

Details on sample attrition across the different assessment waves can be found elsewhere (1–3).

**Text S2**

**Statistical Analysis**

Prior to the main analyses, we calculated descriptive statistics and bivariate correlations for all study variables, including the covariates sex, household SES, migration background, education, gaming experience, and daily tobacco and alcohol use.

We regressed hair substance concentrations as a continuous dose variable and all covariates onto the four CANTAB scores, estimating both bivariate and multivariable associations. When the Breusch–Pagan test indicated nonconstant variance in the model residuals, we applied a White correction to obtain heteroscedasticity robust estimators (4–6). We also estimated the effect sizes (Cohen’s *d*) for each substance by comparing the marginal mean CANTAB scores of participants without any substance concentration to those with a) any substance concentration, and b) low, medium, and high concentrations (i.e., concentration levels), based on a tertile split. Lastly, we conducted robustness checks by including internalizing and externalizing symptoms as additional covariates to adjust for potential confounding of psychiatric symptoms. Notably, these scales were not part of our a priori covariate set but were included post hoc due to their conceptual relevance to both substance use and cognitive outcomes.

All calculations were performed using *R* version 4.3.2 statistical software (7) and the packages *mice* (van Buuren & Groothuis–Oudshoorn, 2011; for imputing missing values), *psych* (Revelle, 2020; for calculating psychometric properties and correlations), *car* (Fox & Sanford, 2019; testing regression models), *fixest* (Berge, 2018; for estimating heteroscedasticity robust standard errors), *emmeans* (Lenth, 2024; for calculating marginal means and estimating effect sizes), and *ggplot2* (Wickham, 2016; for visualizing data and results).

| **Figure S1** Outlier overview in CANTAB data |
| --- |
| 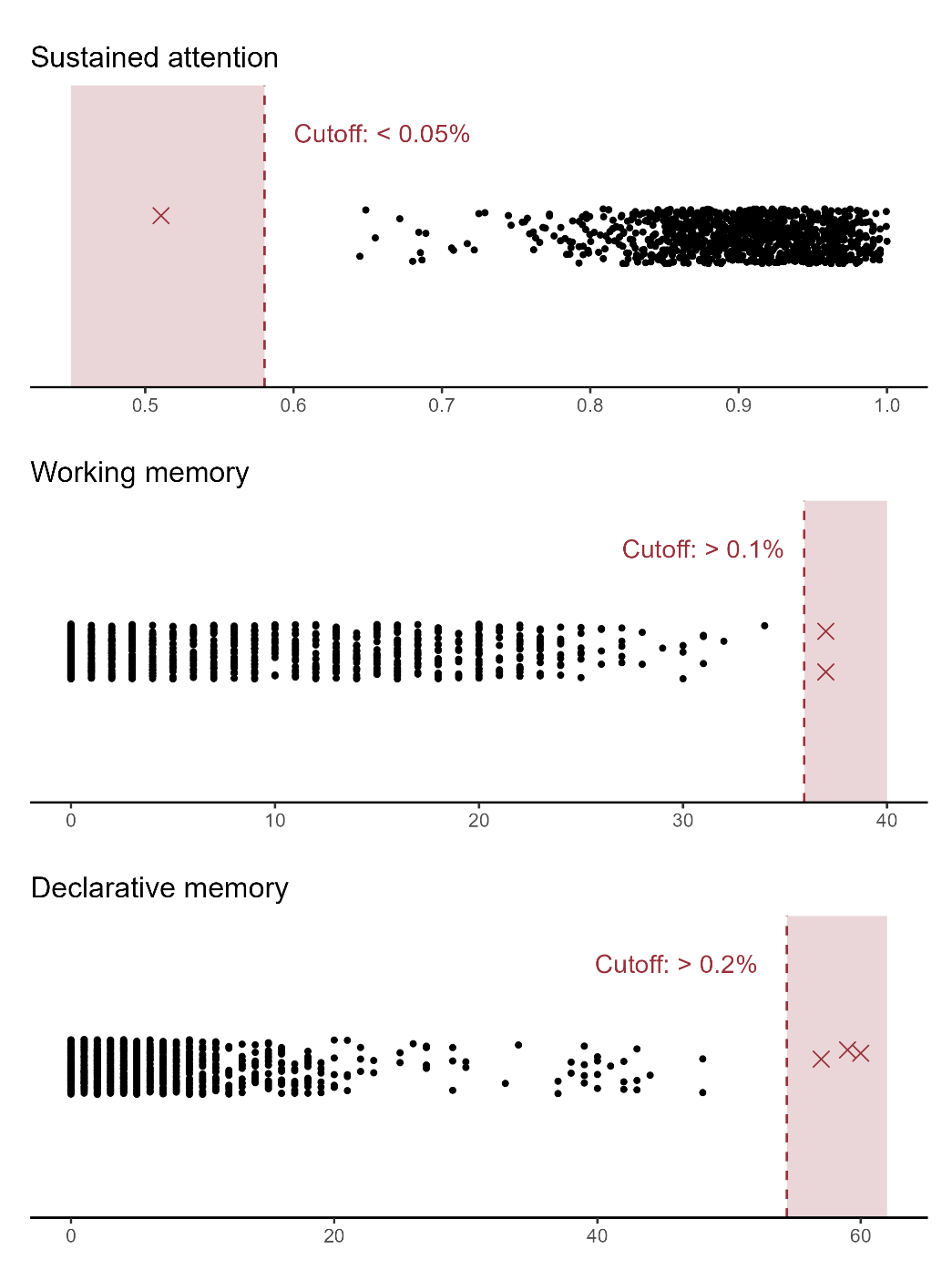 |
| *Note*. For the cutoff, values below the 0.1^st^ percentile were excluded, applying a tolerance factor of 2 (i.e., data between the 0.05^th^ and 0.2^nd^ percentiles) based on visual inspection of the distributions |

| **Figure S2** Gaussian kernel density estimates of transformed study variables |
| --- |
| 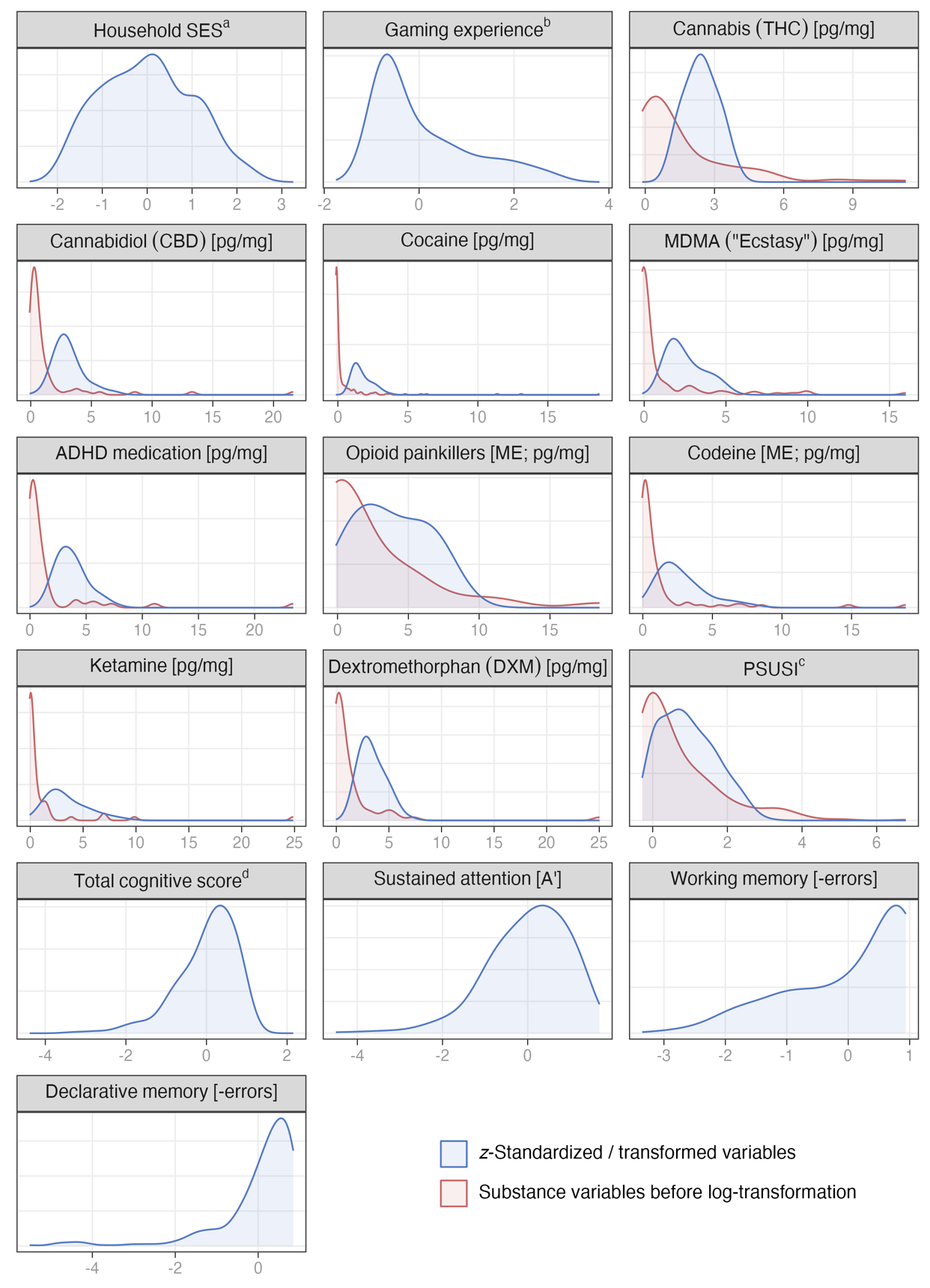 |
| *Note*. All variables were *z*-standardized and substance variables were also log-transformed. [ME] = morphine equivalents. ^a^ Socioeconomic status assessed with the International Socio-Economic Index of Occupational Status (ISEI), ranging from 14 (unskilled worker) to 90 (judge).  ^b^ Frequency of playing action-packed video games in the past year, averaged across ages 20 and 24.  ^c^ Polysubstance-Use Severity Index.  ^e^ Total cognitive score reflects mean score of the three CANTAB scores on sustained attention (*z*-standardized), declarative memory (inverted and *z*-standardized), and working memory (inverted and *z*-standardized). |

| **Figure S3** Bivariate and multivariable associations between substance concentration in hair and total cognitive score |
| --- |
| 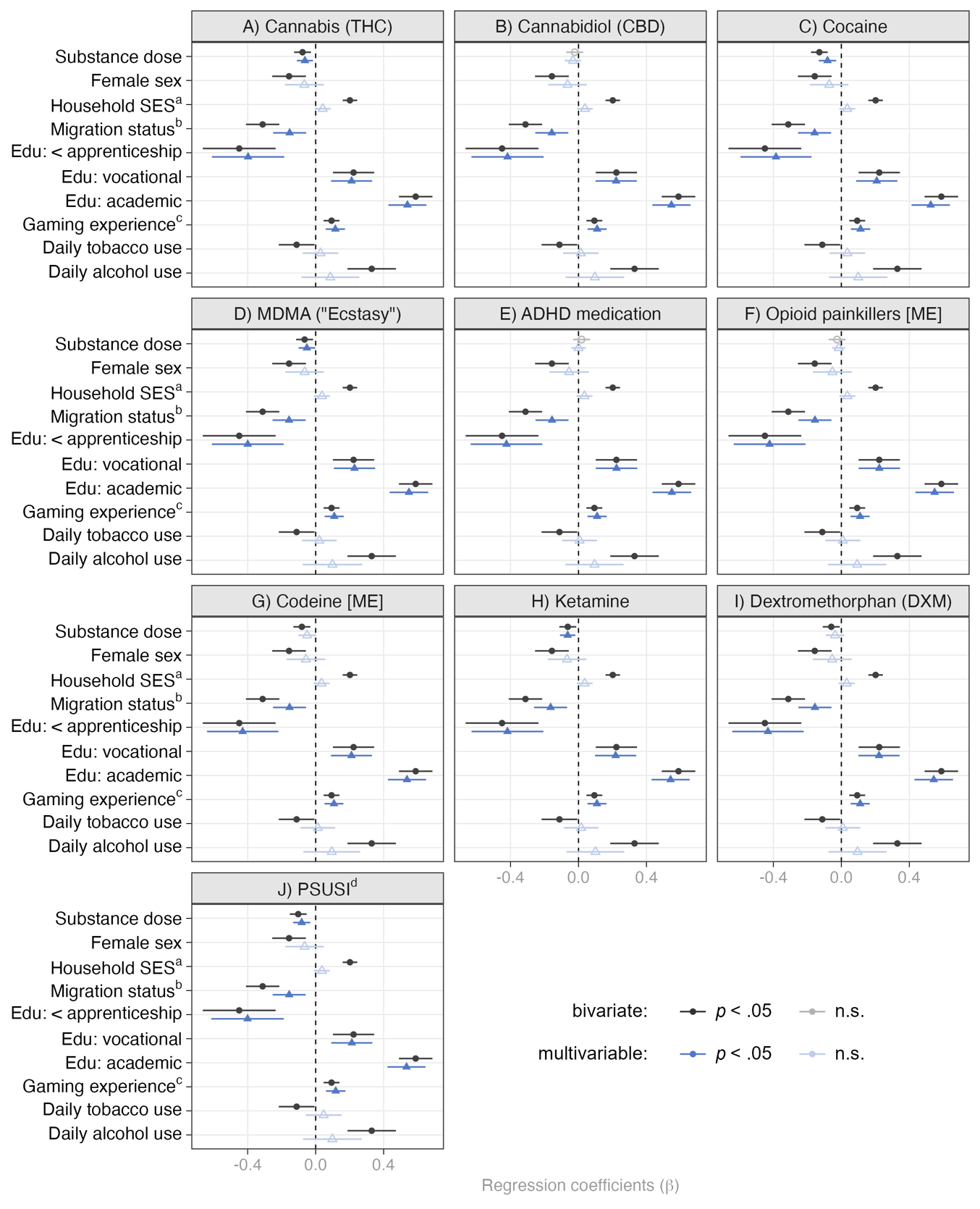 |
| *Note.* Statistically significant results (*p* < .05) are displayed with full saturation. [ME] = morphine equivalents. ^a^ Socioeconomic status assessed with the International Socio-Economic Index of Occupational Status (ISEI), ranging from 14 (unskilled worker) to 90 (judge). ^b^ Positive migration background if both parents were not born in Switzerland. ^c^ Frequency of playing action-packed video games in the past year, averaged across ages 20 and 24.  ^d^ PSUSI = Polysubstance-Use Severity Index |

| **Figure S4** Bivariate and multivariable associations between substance concentration in hair and sustained attention |
| --- |
| 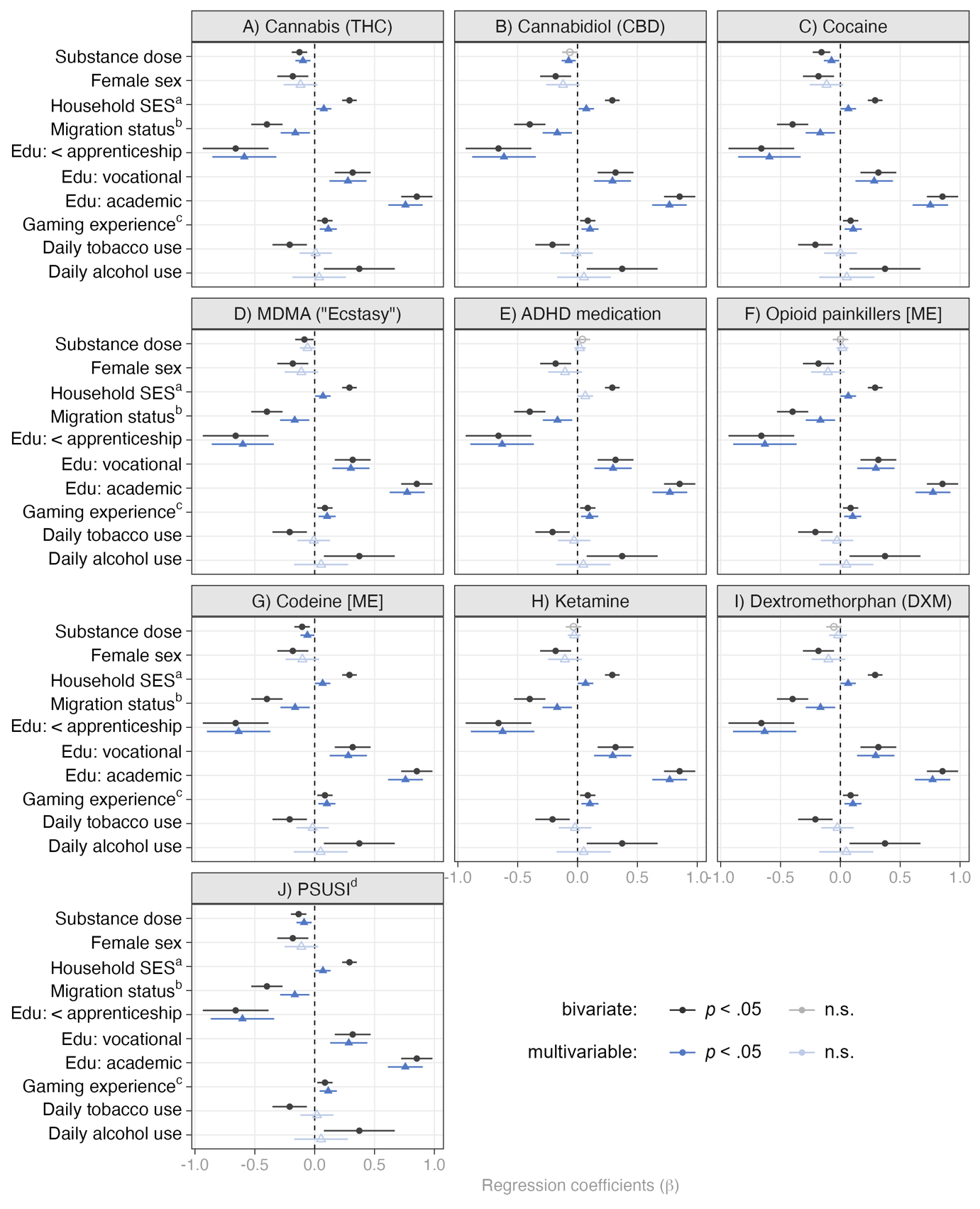 |
| *Note.* Statistically significant results (*p* < .05) are displayed with full saturation. [ME] = morphine equivalents. ^a^ Socioeconomic status assessed with the International Socio-Economic Index of Occupational Status (ISEI), ranging from 14 (unskilled worker) to 90 (judge). ^b^ Positive migration background if both parents were not born in Switzerland. ^c^ Frequency of playing action-packed video games in the past year, averaged across ages 20 and 24.  ^d^ PSUSI = Polysubstance-Use Severity Index |

| **Figure S5** Bivariate and multivariable associations between substance concentration in hair and working memory |
| --- |
| 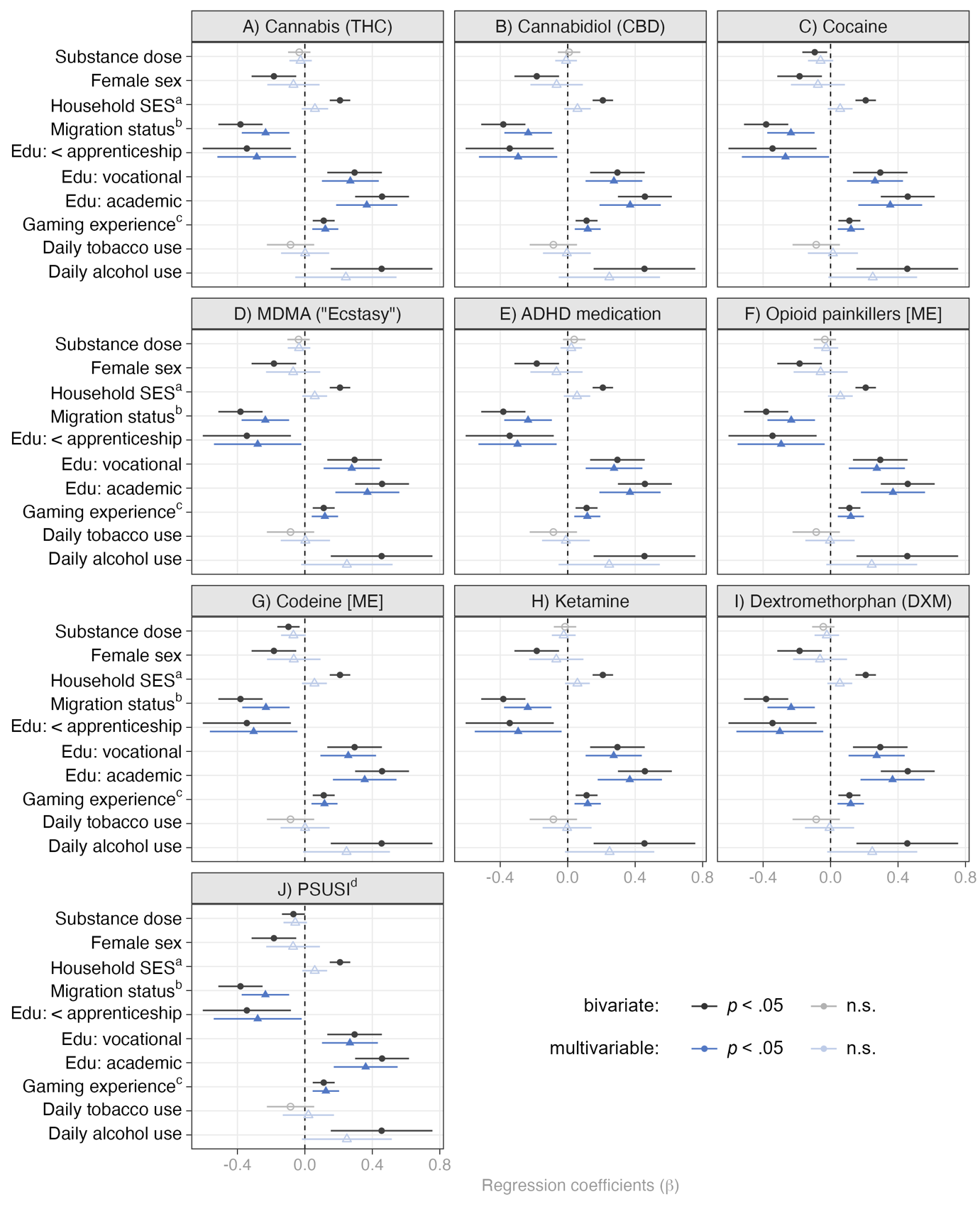 |
| *Note.* Statistically significant results (*p* < .05) are displayed with full saturation. [ME] = morphine equivalents. ^a^ Socioeconomic status assessed with the International Socio-Economic Index of Occupational Status (ISEI), ranging from 14 (unskilled worker) to 90 (judge). ^b^ Positive migration background if both parents were not born in Switzerland. ^c^ Frequency of playing action-packed video games in the past year, averaged across ages 20 and 24.  ^d^ PSUSI = Polysubstance-Use Severity Index |

| **Figure S6** Bivariate and multivariable associations between substance concentration in hair and declarative memory |
| --- |
| 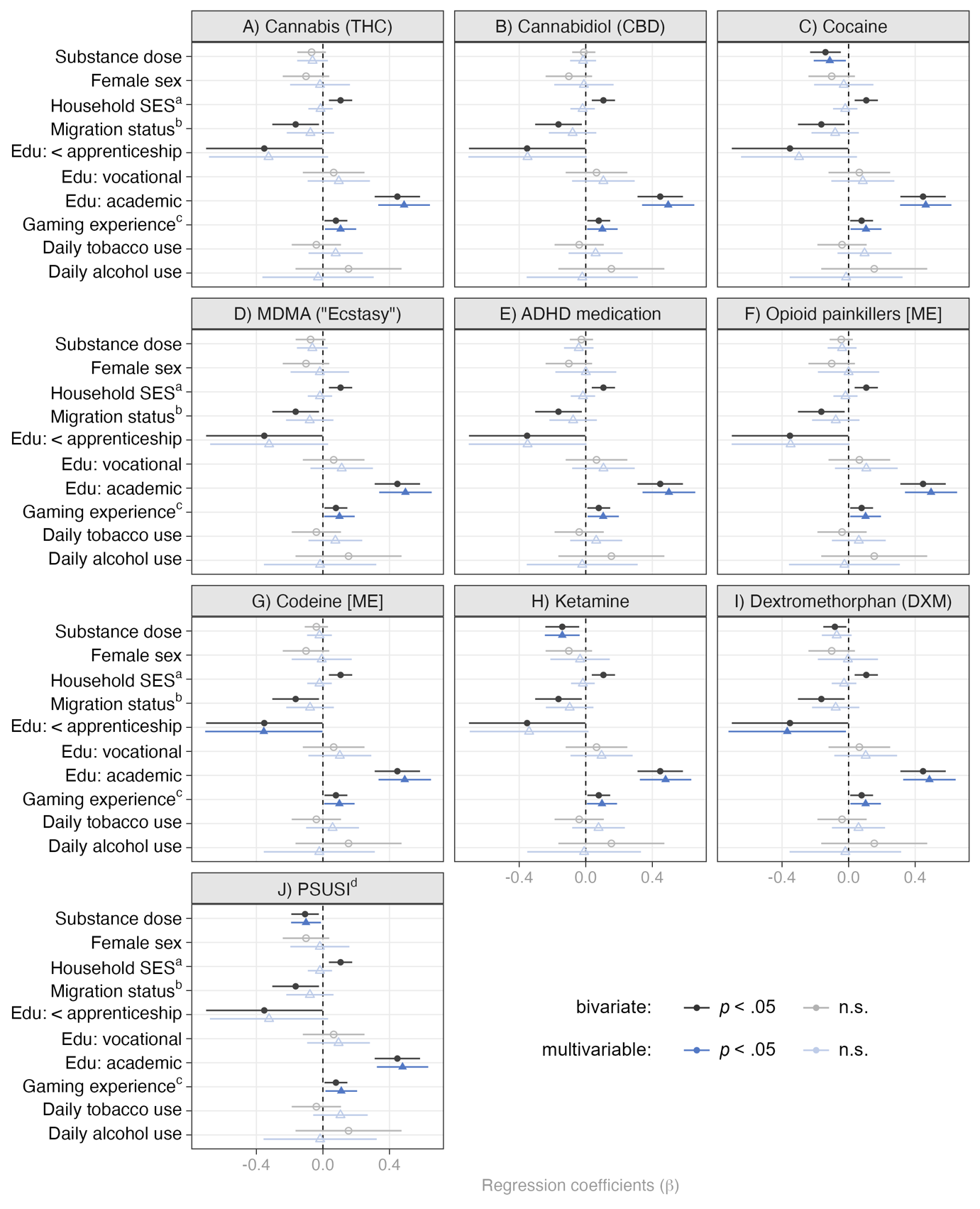 |
| *Note.* Statistically significant results (*p* < .05) are displayed with full saturation. [ME] = morphine equivalents. ^a^ Socioeconomic status assessed with the International Socio-Economic Index of Occupational Status (ISEI), ranging from 14 (unskilled worker) to 90 (judge). ^b^ Positive migration background if both parents were not born in Switzerland. ^c^ Frequency of playing action-packed video games in the past year, averaged across ages 20 and 24.  ^d^ PSUSI = Polysubstance-Use Severity Index |

| **Figure S7** Sensitivity Analyses Controlling for Internalizing and Externalizing Symptoms. |
| --- |
| 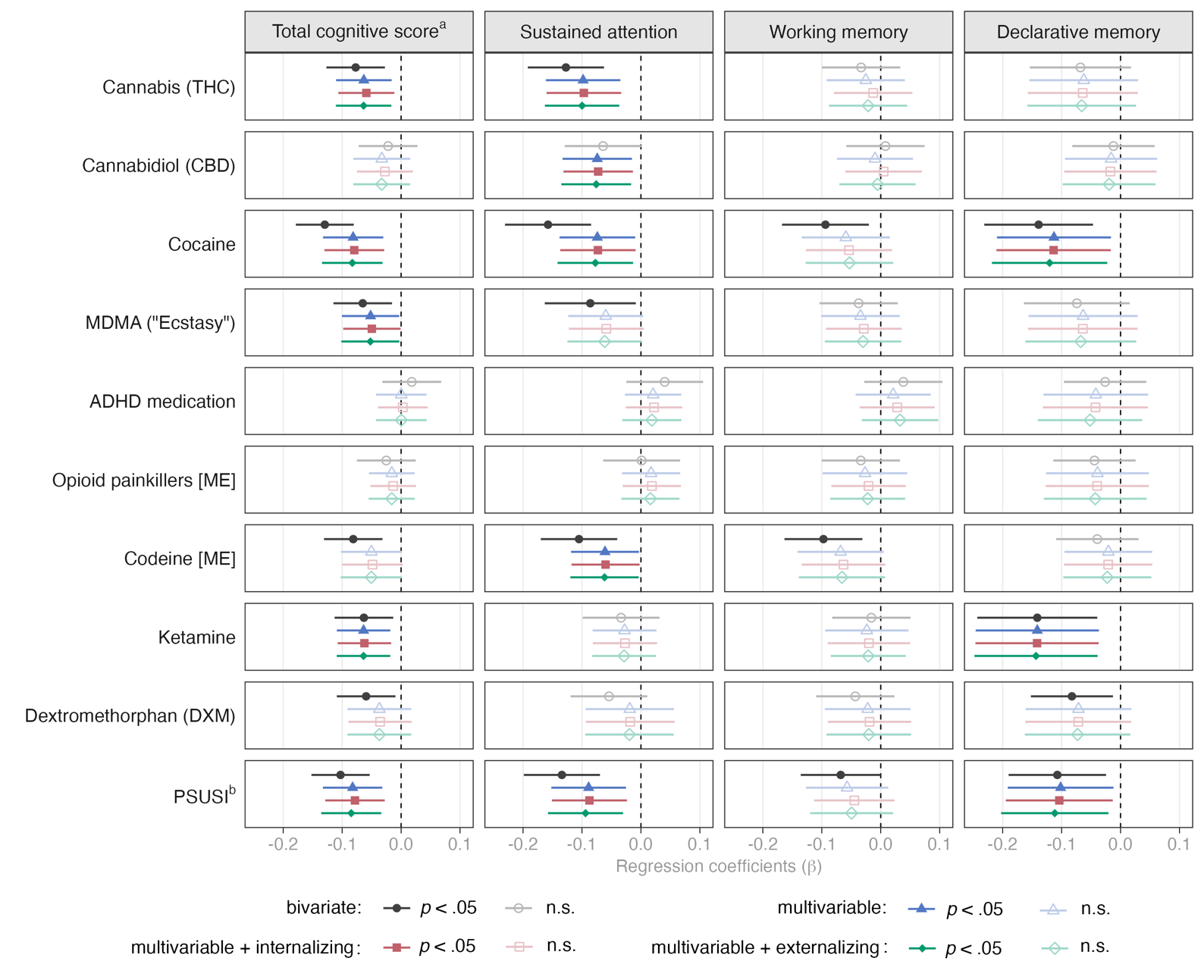 |
| *Note.* Multivariable models include sex, household SES, migration background, education, gaming experience, daily tobacco use, and daily alcohol use as control variables. [ME] = morphine equivalents. Internalizing and Externalizing Symptoms were measured using the Social Behavior Questionnaire (14).  ^a^ Total cognitive score reflects mean score of the three CANTAB scores on sustained attention (*z*-standardized), declarative memory (inverted and *z*-standardized), and working memory (inverted and *z*-standardized).  ^b^ Polysubstance-Use Severity Index. |

| **Table S1** List of covariates and how they were computed from the content of the initial questions (Q), corresponding answer coding (A), and assessment wave (W) | |
| --- | --- |
| **Sex** | |
| Q*:* | Information on participant’s sex; obtained from primary caregiver |
| A: | 1 = *male*; 2 = *female* |
| W: | Wave 1 (age 7) |
| **Household SES** | |
| Q: | Information on participant’s primary caregivers’ employment, which was subsequently mapped onto the Socio-Economic Index of Occupational Status (ISEI); obtained from participant |
| A: | Ranges from 16 = *unskilled worker* to 90 = *judge* |
| W: | Coalesced across wave 4, 5 and 6 (ages 11, 13, and 15) |
| **Migration background** | |
| Q: | Information on country of birth of participant’s biological mother and father; obtained from participant |
| A: | 0 = *at least one parent born in Switzerland*; 1 = *both parents not born in Switzerland* (original answer coding was 1 = *Switzerland*; 2 = *a different country, namely: [free text option]*) |
| W: | Coalesced across wave 5 and 6 (ages 13 and 15) |
| **Gaming experience** | |
| Q: | Information on participant’s time spent playing “action-packed computer or video games, in which opponents are realistically killed or blood is depicted ("shooter" games, etc.)” in the last 12 months; obtained from participant |
| A: | Ranges from 1 = *never* to 7 = *daily* |
| W: | Averaged across wave 8 and 9 (ages 20 and 24) |
| **Daily tobacco use** | |
| Q: | Information on the frequency of participant’s tobacco use in the past three months; obtained from participant |
| A: | 0 = *none or less than daily use*; 1 = *daily use* (original answer coding ranged from 1 = *never* to 5 = *daily*) |
| W: | Wave 9 (age 24) |
| **Daily alcohol use** | |
| Q: | Information on the frequency of participant’s alcohol use in the past three months; obtained from participant |
| A: | 0 = *none or less than daily use*; 1 = *daily use* (original answer coding ranged from 1 = *never* to 5 = *daily*) |
| W: | Wave 9 (age 24) |
|  | |

| **Table S2**  Substances, metabolites, and morphine equivalency (ME) factors | | |
| --- | --- | --- |
| **Substance category** | **Substances and metabolites** | **ME factor** |
| Cannabis (THC) | Tetrahydrocannabinol (THC) |  |
|  | Cannabinol (CBN)^a^ |  |
| Cannabidiol (CBD) | Cannabidiol (CBD) |  |
| Cocaine | Cocaine |  |
|  | Benzoylecgonine^a^ |  |
|  | Norcocaine^a^ |  |
|  | Cocaethylene^a^ |  |
|  | *m*-Hydroxycocaine^a^ |  |
|  | *p*-Hydroxycocaine^a^ |  |
| MDMA („Ecstasy“) | 3,4-Methylenedioxymethamphetamine (MDMA) |  |
|  | 3,4-Methylenedioxyamphetamine (MDA)^a^ |  |
| ADHD medication | Methylphenidate |  |
| Opioid painkillers [ME] | Morphine | 1.000 |
|  | Fentanyl | 128.333 |
|  | Pethidine | 0.125 |
|  | Tapentadol | 0.275 |
|  | Tramadol | 0.116 |
|  | *n*-Desmethyltramadol^a^ | 0.116 |
|  | Methadone | 2.800 |
|  | 2-Ethylidene-1,5-dimethyl-3,3-diphenylpyrrolidine (EDDP)^a^ | 2.800 |
|  | Oxycodone | 1.663 |
|  | Noroxycodone^a^ | 1.663 |
| Codeine [ME] | Codeine | 0.145 |
|  | Dihydrocodeine | 0.231 |
| Ketamine | Ketamine |  |
|  | Norketamine^a^ |  |
| Dextromethorphan (DXM) | Dextromethorphan (DXM) |  |
| *Note*. Morphine equivalency (ME) factors were adopted courtesy of Kroll et al. (2018) and Nissen et al. (2001). ^a^ metabolite | | |

| **Table S3** Bivariate Pearson correlation coefficients for cognition and covariates | | | | | | | | | | |
| --- | --- | --- | --- | --- | --- | --- | --- | --- | --- | --- |
|  | 1. | 2. | 3. | 4. | 5. | 6. | 7. | 8. | 9. | 10. |
| 1. Female sex | – |  |  |  |  |  |  |  |  |  |
| 2. Household SES^a^ | -.03 | – |  |  |  |  |  |  |  |  |
| 3. Migration background^b^ | -.02 | **-.42^***^** | – |  |  |  |  |  |  |  |
| 4. Completed education | .04 | **.46^***^** | **-.20^***^** | – |  |  |  |  |  |  |
| 5. Gaming experience^c^ | **-.57^***^** | .00 | -.03 | **-.13^***^** | – |  |  |  |  |  |
| 6. Daily tobacco use | -.05 | **-.09^**^** | **.07^*^** | **-.23^***^** | **.09^**^** | – |  |  |  |  |
| 7. Daily alcohol use | -.05 | **.15^***^** | **-.09^*^** | **.13^***^** | .00 | **.20^***^** | – |  |  |  |
| 8. Total cognitive score^d^ | **-.11^**^** | **.27^***^** | **-.21^***^** | **.41^***^** | **.13^***^** | **-.07^*^** | **.10^**^** | – |  |  |
| 9. Sustained attention | **-.09^**^** | **.30^***^** | **-.21^***^** | **.46^***^** | **.09^**^** | **-.10^**^** | **.08^*^** | **.75^***^** | – |  |
| 10. Declarative memory | -.05 | **.10^**^** | **-.08^*^** | **.22^***^** | **.08^*^** | -.02 | .03 | **.74^***^** | **.33^***^** | – |
| 11. Working memory | **-.09^**^** | **.21^***^** | **-.19^***^** | **.25^***^** | **.11^***^** | -.04 | **.10^**^** | **.73^***^** | **.35^***^** | **.27^***^** |
| *Note.* Bold formatting indicates statistical significance at an alpha-level of .05  ^*^ *p* < .05, ^**^ *p* < .01, ^***^ *p* < .001  ^a^ Socioeconomic status assessed with the International Socio-Economic Index of Occupational Status (ISEI), ranging from 14 (unskilled worker) to 90 (judge). ^b^ Positive migration background if both parents were not born in Switzerland. ^c^ Frequency of playing action-packed video games in the past year, averaged across ages 20 and 24.  ^d^ Total cognitive score reflects mean score of the three CANTAB scores on sustained attention (*z*-standardized), declarative memory (inverted and *z*-standardized), and working memory (inverted and *z*-standardized). | | | | | | | | | | |

| **Table S4**  Descriptives of raw (A) and transformed (B) study variables | | | | | | | |
| --- | --- | --- | --- | --- | --- | --- | --- |
|  | ***n*** | **Mean (*SD*)** | **Median (*MAD*)** | **Min.** | **Max.** | **Skew** | **Kurtosis** |
| **A) Raw variables** | | | | | | | |
| **Sociodemographics** |  |  |  |  |  |  |  |
| Household SES^a^ | 850 | 46.9 (19.0) | 48.0 (23.7) | 16.0 | 90.0 | 0.2 | -0.8 |
| Gaming experience^b^ | 850 | 2.4 (1.7) | 1.5 (0.7) | 1.0 | 7.0 | 1.1 | 0.2 |
| **Substances in hair (pg/mg)** |  |  |  |  |  |  |  |
| Cannabis (THC) | 116 | 159.9 (191.2) | 90.0 (91.9) | 10.0 | 1030.0 | 2.1 | 5.2 |
| Cannabidiol (CBD) | 71 | 40.1 (88.0) | 14.0 (11.9) | 1.0 | 600.0 | 4.6 | 23.4 |
| Cocaine | 197 | 1645.3 (5293.4) | 157.0 (162.0) | 18.7 | 49660.0 | 6.2 | 44.5 |
| MDMA (“Ecstasy”) | 101 | 460.5 (982.2) | 54.0 (57.8) | 10.0 | 5950.0 | 3.1 | 10.9 |
| ADHD medication | 52 | 113.8 (254.0) | 29.5 (27.4) | 5.0 | 1600.0 | 4.3 | 20.5 |
| Opioid painkillers [ME] | 40 | 153.6 (253.8) | 19.6 (27.9) | 0.1 | 1179.8 | 2.2 | 5.1 |
| Codeine [ME] | 75 | 15.3 (33.1) | 3.9 (3.6) | 0.9 | 203.3 | 3.9 | 16.5 |
| Ketamine | 53 | 97.7 (280.4) | 11.0 (10.4) | 2.0 | 1830.0 | 4.8 | 25.2 |
| Dextromethorphan (DXM) | 61 | 127.2 (289.0) | 32.0 (32.6) | 6.0 | 2100.0 | 5.4 | 32.8 |
| PSUSI^c^ | 391 | 4.1 (3.5) | 3.0 (3.0) | 1.0 | 23.0 | 1.7 | 3.4 |
| **CANTAB^d^** |  |  |  |  |  |  |  |
| Sustained attention | 850 | 0.9 (0.1) | 0.9 (0.1) | 0.6 | 1.0 | -0.9 | 1.6 |
| Working memory | 849 | 7.2 (7.8) | 4.0 (5.9) | 0.0 | 34.0 | 0.9 | -0.2 |
| Declarative memory | 848 | 6.4 (7.8) | 4.0 (4.4) | 0.0 | 48.0 | 2.6 | 8.4 |
| **B) Transformed variables** | | | | | | | |
| **Sociodemographics** |  |  |  |  |  |  |  |
| Household SES^a^ | 850 | 0.0 (1.0) | 0.1 (1.2) | -1.6 | 2.3 | 0.2 | -0.8 |
| Gaming experience^b^ | 850 | 0.0 (1.0) | -0.5 (0.4) | -0.8 | 2.8 | 1.1 | 0.2 |
| **Substances in hair (pg/mg)** |  |  |  |  |  |  |  |
| Cannabis (THC) | 116 | 2.4 (0.7) | 2.4 (0.8) | 1.1 | 4.0 | 0.1 | -0.9 |
| Cannabidiol (CBD) | 71 | 3.1 (1.2) | 2.8 (1.1) | 0.5 | 7.1 | 1.0 | 1.1 |
| Cocaine | 197 | 1.7 (0.7) | 1.5 (0.6) | 0.7 | 3.8 | 0.9 | 0.0 |
| MDMA (“Ecstasy”) | 101 | 2.5 (1.1) | 2.2 (1.1) | 1.2 | 5.2 | 0.7 | -0.7 |
| ADHD medication | 52 | 3.7 (1.4) | 3.4 (1.3) | 1.7 | 7.6 | 0.8 | 0.2 |
| Opioid painkillers [ME] | 40 | 3.8 (2.5) | 3.4 (3.4) | -0.1 | 8.2 | 0.1 | -1.4 |
| Codeine [ME] | 75 | 2.8 (1.7) | 2.2 (1.4) | 0.7 | 8.0 | 1.2 | 0.9 |
| Ketamine | 53 | 3.4 (1.9) | 2.8 (1.6) | 1.1 | 8.9 | 1.1 | 0.2 |
| Dextromethorphan (DXM) | 61 | 3.4 (1.2) | 3.0 (1.3) | 1.6 | 7.0 | 0.7 | -0.2 |
| PSUSI^c^ | 391 | 0.9 (0.7) | 0.9 (0.7) | 0.0 | 3.1 | 0.5 | -0.7 |
| **CANTAB^d^** |  |  |  |  |  |  |  |
| Total cognitive score^e^ | 850 | 0.0 (0.7) | 0.2 (0.6) | -3.4 | 1.2 | -1.2 | 2.0 |
| Sustained attention | 850 | 0.0 (1.0) | 0.1 (1.0) | -4.5 | 1.7 | -0.9 | 1.6 |
| Working memory | 849 | 0.0 (1.0) | 0.4 (0.7) | -3.4 | 0.9 | -0.9 | -0.2 |
| Declarative memory | 848 | 0.0 (1.0) | 0.3 (0.6) | -5.5 | 0.8 | -2.6 | 8.4 |
| *Note*. In B), all variables were *z*-standardized, working and declarative memory variables were additionally inverted, and substance variables were additionally log-transformed. [ME] = morphine equivalents. *n* = number of complete cases (only positive cases for substance use variables were used). *SD* = standard deviation. *MAD* = median absolute deviation.  ^a^ Socioeconomic status assessed with the International Socio-Economic Index of Occupational Status (ISEI), ranging from 14 (unskilled worker) to 90 (judge). ^b^ Frequency of playing action-packed video games in the past year, averaged across ages 20 and 24. ^c^ Polysubstance-Use Severity Index.  ^d^ CANTAB = Cambridge Neuropsychological Test Automated Battery.  ^e^ Total cognitive score reflects mean score of the three CANTAB scores on sustained attention (*z*-standardized), declarative memory (inverted and *z*-standardized), and working memory (inverted and *z*-standardized). | | | | | | | |

| **Table S5**  Marginal effects (Cohen’s *d*) of any, low, medium, and high substance concentrations on cognitive functions | | | | | | | | | |
| --- | --- | --- | --- | --- | --- | --- | --- | --- | --- |
|  |  | **Total cognitive score^a^** | | **Sustained attention** | | **Working memory** | | **Declarative memory** | |
|  | *n* (%) | *d* | 95%-*CI* | *d* | 95%-*CI* | *d* | 95%*CI* | *d* | 95%-*CI* |
| Cannabis (THC) | | | | | | | | | |
| any | 116 (13.6) | **0.31** | [0.11; 0.52] | **0.40** | [0.19; 0.61] | 0.08 | [-0.13; 0.28] | 0.20 | [-0.01; 0.41] |
| low | 40 (4.7) | **0.43** | [0.10; 0.75] | **0.63** | [0.31; 0.96] | 0.06 | [-0.27; 0.38] | 0.25 | [-0.07; 0.58] |
| medium | 37 (4.4) | 0.24 | [-0.10; 0.58] | 0.14 | [-0.20; 0.48] | 0.04 | [-0.30; 0.38] | 0.28 | [-0.06; 0.62] |
| high | 39 (4.6) | 0.27 | [-0.07; 0.60] | **0.40** | [0.07; 0.73] | 0.14 | [-0.20; 0.47] | 0.07 | [-0.27; 0.40] |
| Cannabidiol (CBD) | | | | | | | | | |
| any | 71 (8.4) | 0.19 | [-0.06; 0.44] | **0.29** | [0.04; 0.54] | 0.02 | [-0.23; 0.28] | 0.11 | [-0.14; 0.36] |
| low | 25 (2.9) | 0.24 | [-0.16; 0.65] | 0.24 | [-0.17; 0.65] | 0.08 | [-0.33; 0.48] | 0.21 | [-0.20; 0.61] |
| medium | 22 (2.6) | 0.10 | [-0.33; 0.53] | 0.05 | [-0.38; 0.47] | 0.03 | [-0.40; 0.46] | 0.14 | [-0.29; 0.57] |
| high | 24 (2.8) | 0.21 | [-0.20; 0.62] | **0.56** | [0.15; 0.97] | 0.03 | [-0.38; 0.45] | 0.02 | [-0.39; 0.43] |
| Cocaine | | | | | | | | | |
| any | 197 (23.2) | **0.24** | [0.07; 0.41] | **0.21** | [0.04; 0.38] | 0.10 | [-0.06; 0.27] | **0.20** | [0.04; 0.37] |
| low | 66 (7.8) | 0.20 | [-0.06; 0.46] | 0.23 | [-0.03; 0.49] | 0.09 | [-0.16; 0.35] | 0.12 | [-0.14; 0.37] |
| medium | 65 (7.6) | 0.01 | [-0.25; 0.28] | 0.00 | [-0.26; 0.27] | 0.03 | [-0.23; 0.29] | 0.05 | [-0.21; 0.32] |
| high | 66 (7.8) | **0.52** | [0.26; 0.79] | **0.40** | [0.13; 0.66] | 0.25 | [-0.01; 0.52] | **0.45** | [0.19; 0.72] |
| MDMA (“Ecstasy”) | | | | | | | | | |
| any | 101 (11.9) | **0.25** | [0.03; 0.46] | **0.25** | [0.04; 0.47] | 0.13 | [-0.08; 0.35] | 0.16 | [-0.05; 0.37] |
| low | 35 (4.1) | 0.16 | [-0.18; 0.51] | 0.21 | [-0.13; 0.56] | 0.22 | [-0.12; 0.56] | 0.05 | [-0.29; 0.40] |
| medium | 32 (3.8) | **0.40** | [0.03; 0.76] | **0.53** | [0.17; 0.89] | 0.15 | [-0.21; 0.51] | 0.20 | [-0.16; 0.56] |
| high | 34 (4.0) | 0.20 | [-0.14; 0.55] | 0.04 | [-0.31; 0.39] | 0.03 | [-0.32; 0.38] | 0.34 | [-0.01; 0.69] |
| ADHD medication | | | | | | | | | |
| any | 52 (6.1) | 0.04 | [-0.24; 0.32] | 0.09 | [-0.19; 0.38] | 0.09 | [-0.19; 0.38] | 0.24 | [-0.04; 0.53] |
| low | 18 (2.1) | 0.32 | [-0.15; 0.79] | 0.05 | [-0.42; 0.52] | 0.24 | [-0.23; 0.71] | 0.45 | [-0.02; 0.92] |
| medium | 17 (2.0) | 0.36 | [-0.13; 0.84] | 0.02 | [-0.47; 0.51] | **0.55** | [0.06; 1.03] | 0.17 | [-0.31; 0.66] |
| high | 17 (2.0) | 0.13 | [-0.35; 0.61] | 0.21 | [-0.27; 0.70] | 0.00 | [-0.48; 0.48] | 0.44 | [-0.05; 0.92] |
| Opioid painkillers [ME] | | | | | | | | | |
| any | 40 (4.7) | 0.08 | [-0.24; 0.40] | 0.05 | [-0.27; 0.37] | 0.13 | [-0.19; 0.45] | 0.09 | [-0.23; 0.41] |
| low | 14 (1.6) | 0.03 | [-0.51; 0.57] | 0.12 | [-0.41; 0.66] | 0.00 | [-0.53; 0.54] | 0.04 | [-0.49; 0.58] |
| medium | 13 (1.5) | 0.01 | [-0.54; 0.57] | 0.01 | [-0.55; 0.56] | 0.07 | [-0.48; 0.63] | 0.04 | [-0.51; 0.60] |
| high | 13 (1.5) | 0.21 | [-0.34; 0.76] | 0.30 | [-0.26; 0.85] | 0.31 | [-0.24; 0.86] | 0.37 | [-0.18; 0.92] |
| Codeine [ME] | | | | | | | | | |
| any | 75 (8.8) | **0.25** | [0.01; 0.49] | 0.22 | [-0.02; 0.46] | **0.29** | [0.05; 0.53] | 0.03 | [-0.21; 0.27] |
| low | 26 (3.1) | 0.31 | [-0.08; 0.71] | 0.20 | [-0.20; 0.59] | **0.48** | [0.08; 0.87] | 0.04 | [-0.36; 0.45] |
| medium | 24 (2.8) | 0.02 | [-0.39; 0.43] | 0.08 | [-0.33; 0.49] | 0.01 | [-0.40; 0.42] | 0.04 | [-0.37; 0.45] |
| high | 25 (2.9) | **0.45** | [0.05; 0.86] | **0.54** | [0.13; 0.94] | 0.37 | [-0.03; 0.77] | 0.10 | [-0.30; 0.50] |
| Ketamine | | | | | | | | | |
| any | 53 (6.2) | **0.42** | [0.13; 0.70] | 0.21 | [-0.07; 0.50] | 0.06 | [-0.23; 0.34] | **0.59** | [0.31; 0.88] |
| low | 20 (2.4) | **0.61** | [0.16; 1.06] | 0.43 | [-0.02; 0.88] | 0.02 | [-0.43; 0.47] | **0.82** | [0.37; 1.27] |
| medium | 15 (1.8) | 0.15 | [-0.37; 0.67] | 0.09 | [-0.42; 0.61] | 0.05 | [-0.46; 0.57] | 0.27 | [-0.24; 0.79] |
| high | 18 (2.1) | 0.43 | [-0.04; 0.90] | 0.08 | [-0.39; 0.55] | 0.19 | [-0.29; 0.66] | **0.61** | [0.14; 1.08] |
| Dextromethorphan (DXM) | | | | | | | | | |
| any | 61 (7.2) | 0.19 | [-0.07; 0.45] | 0.03 | [-0.23; 0.30] | 0.13 | [-0.13; 0.40] | **0.28** | [0.02; 0.55] |
| low | 22 (2.6) | 0.05 | [-0.38; 0.48] | 0.25 | [-0.18; 0.67] | 0.12 | [-0.31; 0.54] | 0.20 | [-0.22; 0.63] |
| medium | 19 (2.2) | 0.45 | [-0.01; 0.91] | 0.07 | [-0.39; 0.53] | 0.41 | [-0.05; 0.87] | **0.56** | [0.10; 1.02] |
| high | 20 (2.4) | 0.10 | [-0.35; 0.55] | 0.24 | [-0.21; 0.69] | 0.11 | [-0.34; 0.56] | 0.10 | [-0.35; 0.55] |
| PSUSI^b^ | | | | | | | | | |
| any | 391 (46.0) | **0.21** | [0.07; 0.35] | **0.17** | [0.03; 0.32] | **0.15** | [0.01; 0.29] | 0.12 | [-0.02; 0.26] |
| low | 169 (19.9) | 0.11 | [-0.07; 0.29] | 0.06 | [-0.11; 0.24] | 0.15 | [-0.02; 0.33] | 0.02 | [-0.16; 0.20] |
| medium | 94 (11.1) | **0.32** | [0.10; 0.55] | **0.36** | [0.13; 0.59] | 0.17 | [-0.06; 0.40] | 0.16 | [-0.07; 0.39] |
| high | 128 (15.1) | **0.27** | [0.07; 0.48] | 0.20 | [0.00; 0.41] | 0.12 | [-0.08; 0.33] | **0.26** | [0.05; 0.46] |
| Note. Effect sizes (Cohen’s *d*) and 95% confidence intervals (*CI*) were estimated by marginal mean differences across the covariates sex, household SES, migration background, education, gaming experience, daily tobacco use, and daily alcohol use. Bold formatting indicates statistically significant effect sizes at an α-level of .05. [ME] = morphine equivalents.  ^a^ Total cognitive score reflects mean score of the three CANTAB scores on sustained attention (*z-*standardized), declarative memory (inverted and *z-*standardized), and working memory (inverted and *z-*standardized).  ^b^ Polysubstance Use Severity Index. | | | | | | | | | |

| **Table S6**  Percentage of non-users and users scoring 1.5 or 2 standard deviations (SD) below the average cognitive scores | | | | | | | | | |
| --- | --- | --- | --- | --- | --- | --- | --- | --- | --- |
| **Substance** | **Threshold** | **Total cognitive score^b^** | | **Sustained attention** | | **Working memory** | | **Declarative memory** | |
|  |  | Non-users (%) | Users (%) | Non-users (%) | Users (%) | Non-users (%) | Users (%) | Non-users (%) | Users (%) |
| Cannabis (THC) | < 1.5 SD | 6.4 | 12.1 | 6.0 | 11.2 | 11.4 | 12.9 | 5.2 | 10.3 |
|  | < 2.0 SD | 4.4 | 9.5 | 3.4 | 6.9 | 4.9 | 6.0 | 3.1 | 9.5 |
| Cannabidiol (CBD) | < 1.5 SD | 6.9 | 9.9 | 6.2 | 12.7 | 11.7 | 11.3 | 5.6 | 8.5 |
|  | < 2.0 SD | 4.9 | 7.0 | 3.5 | 8.5 | 5.0 | 5.6 | 3.6 | 8.5 |
| Cocaine | < 1.5 SD | 6.1 | 10.7 | 5.8 | 9.6 | 9.8 | 17.8 | 4.9 | 9.1 |
|  | < 2.0 SD | 4.1 | 8.1 | 2.9 | 7.1 | 4.1 | 8.1 | 3.2 | 6.6 |
| MDMA (“Ecstasy”) | < 1.5 SD | 6.4 | 12.9 | 6.1 | 10.9 | 10.7 | 18.8 | 5.1 | 11.9 |
|  | < 2.0 SD | 4.5 | 8.9 | 3.1 | 9.9 | 4.8 | 6.9 | 3.5 | 7.9 |
| ADHD medication | < 1.5 SD | 7.0 | 9.6 | 7.0 | 1.9 | 11.7 | 11.5 | 5.8 | 7.7 |
|  | < 2.0 SD | 4.8 | 9.6 | 4.0 | 1.9 | 5.0 | 5.8 | 3.8 | 7.7 |
| Opioid painkillers [ME] | < 1.5 SD | 7.3 | 5.0 | 6.8 | 5.0 | 11.5 | 15.0 | 5.9 | 5.0 |
|  | < 2.0 SD | 5.1 | 5.0 | 4.0 | 2.5 | 5.2 | 2.5 | 4.0 | 5.0 |
| Codeine [ME] | < 1.5 SD | 6.6 | 13.3 | 6.6 | 8.0 | 10.8 | 20.0 | 5.7 | 8.0 |
|  | < 2.0 SD | 4.5 | 10.7 | 4.0 | 2.7 | 4.6 | 9.3 | 3.6 | 8.0 |
| Ketamine | < 1.5 SD | 6.5 | 17.0 | 6.5 | 9.4 | 11.3 | 17.0 | 4.9 | 20.8 |
|  | < 2.0 SD | 4.6 | 11.3 | 3.6 | 7.5 | 5.0 | 5.7 | 3.4 | 13.2 |
| Dextromethorphan (DXM) | < 1.5 SD | 6.6 | 14.8 | 6.5 | 9.8 | 11.0 | 19.7 | 5.1 | 16.4 |
|  | < 2.0 SD | 4.7 | 9.8 | 3.8 | 4.9 | 4.7 | 9.8 | 3.3 | 13.1 |
| PSUSI^a^ | < 1.5 SD | 5.5 | 13.0 | 5.9 | 9.3 | 10.5 | 15.5 | 4.3 | 11.4 |
|  | < 2.0 SD | 3.7 | 9.8 | 3.2 | 6.2 | 4.6 | 6.7 | 2.6 | 8.8 |
| Note. [ME] = morphine equivalents.  ^a^ Polysubstance-Use Severity Index.  ^b^ Total cognitive score reflects mean score of the three CANTAB scores on sustained attention (*z*-standardized), declarative memory (inverted and *z*-standardized), and working memory (inverted and *z*-standardized). | | | | | | | | | |

**Supplementary References**

1. Eisner NL, Murray AL, Eisner M, Ribeaud D. A practical guide to the analysis of non-response and attrition in longitudinal research using a real data example. Int J Behav Dev [Internet]. 2019 Jan 1 [cited 2024 Jul 5];43(1):24–34. Available from: https://journals.sagepub.com/doi/full/10.1177/0165025418797004

2. Quednow BB, Steinhoff A, Bechtiger L, Ribeaud D, Eisner M, Shanahan L. High Prevalence and Early Onsets: Legal and Illegal Substance Use in an Urban Cohort of Young Adults in Switzerland. Eur Addict Res [Internet]. 2022 May 16 [cited 2024 Jun 27];28(3):186–98. Available from: https://dx.doi.org/10.1159/000520178

3. Steinhoff A, Bechtiger L, Ribeaud D, Eisner MP, Quednow BB, Shanahan L. Polysubstance Use in Early Adulthood: Patterns and Developmental Precursors in an Urban Cohort. Front Behav Neurosci. 2022 Jan 27;15:797473.

4. MacKinnon JG, White H. Some heteroskedasticity-consistent covariance matrix estimators with improved finite sample properties. J Econom. 1985 Sep 1;29(3):305–25.

5. Breusch TS, Pagan AR. A Simple Test for Heteroscedasticity and Random Coefficient Variation. Econometrica [Internet]. 1979;47(5):1287–94. Available from: http://www.jstor.org/stable/1911963

6. Fox J. Applied Regression Analysis and Generalized Linear Models. Third. Sage; 2016.

7. R Core Team. R: A Language and Environment for Statistical Computing [Internet]. Vienna, Austria: R Foundation for Statistical Computing; 2023 [cited 2024 May 17]. Available from: https://www.R-project.org/

8. van Buuren S, Groothuis-Oudshoorn K. mice: Multivariate Imputation  by Chained Equations in R. J Stat Softw. 2011;45(3):1–67.

9. Revelle W. psych: Procedures for personality and psychological research [Internet]. Northwestern University, Evanston, Illinois, USA; 2020. Available from: https://cran.r-project.org/package=psych

10. Fox J, Sanford W. An R Companion to Applied Regression [Internet]. Thousand Oaks CA: Sage; 2019. Available from: https://socialsciences.mcmaster.ca/jfox/Books/Companion/

11. Berge L. Efficient estimation of maximum likelihood models with multiple fixed-effects: the R package FENmlm. CREA Discussion Papers. 2018;13.

12. Lenth R V. emmeans: Estimated Marginal Means, aka Least-Squares Means [Internet]. 2024 [cited 2024 May 17]. Available from: https://CRAN.R-project.org/package=emmeans

13. Wickham H. ggplot2: Elegant graphics for data analysis [Internet]. Springer-Verlag, New York; 2016. Available from: https://ggplot2.tidyverse.org

14. Murray AL, Obsuth I, Eisner M, Ribeaud D. Evaluating Longitudinal Invariance in Dimensions of Mental Health Across Adolescence: An Analysis of the Social Behavior Questionnaire. https://doi.org/101177/1073191117721741 [Internet]. 2017 Jul 30 [cited 2025 Jan 23];26(7):1234–45. Available from: https://journals.sagepub.com/doi/10.1177/1073191117721741
